# Supplementary material for: A Comprehensive Assessment of Ultraviolet-Radiation-Induced Mutations in Flammulina filiformis Using Whole-Genome Resequencing
Source: J Fungi (Basel). 2024 Mar 20;10(3):228. doi: 10.3390/jof10030228 (PMC10971301; doi:10.3390/jof10030228)
Supplement: Supplementary file 1 [file jof-10-00228-s001.zip › Supplementary Material S8/KEGG annotation/out/64381550635650.os/KO/out_map/map04015.html]

KEGG PATHWAY: Rap1 signaling pathway - Reference pathway


|  |  |
| --- | --- |
| **Rap1 signaling pathway - Reference pathway** |  |

[
Pathway menu
| Organism menu
| Pathway entry
| Show description
| User data mapping
]

Rap1 is a small GTPase that controls diverse processes, such as cell adhesion, cell-cell junction formation and cell polarity. Like all G proteins, Rap1 cycles between an inactive GDP-bound and an active GTP-bound conformation. A variety of extracellular signals control this cycle through the regulation of several unique guanine nucleotide exchange factors (GEFs) and GTPase activating proteins (GAPs). Rap1 plays a dominant role in the control of cell-cell and cell-matrix interactions by regulating the function of integrins and other adhesion molecules in various cell types. Rap1 also regulates MAP kinase (MAPK) activity in a manner highly dependent on the context of cell types.

|  |  |
| --- | --- |
| Reference pathway | 100% |
